# Efficacy of the New Neuraminidase Inhibitor CS-8958 against H5N1 Influenza Viruses

**DOI:** 10.1371/journal.ppat.1000786

**Published:** 2010-02-26

**Authors:** Maki Kiso, Shuku Kubo, Makoto Ozawa, Quynh Mai Le, Chairul A. Nidom, Makoto Yamashita, Yoshihiro Kawaoka

**Affiliations:** 1 Division of Virology, Department of Microbiology and Immunology, Institute of Medical Science, University of Tokyo, Shirokanedai, Minato-ku, Tokyo, Japan; 2 Biological Research Laboratories, Daiichi Sankyo Co. Ltd., Hiromachi, Shinagawa-ku, Tokyo, Japan; 3 International Research Center for Infectious Diseases, Institute of Medical Science, University of Tokyo, Shirokanedai, Minato-ku, Tokyo, Japan; 4 Department of Pathobiological Sciences, University of Wisconsin, Madison, Wisconsin, United States of America; 5 National Institute of Hygiene and Epidemiology, Hanoi, Vietnam; 6 Faculty of Veterinary Medicine, Tropical Disease Centre, Airlangga University, Surabaya, Indonesia; 7 ERATO Infection-Induced Host Responses Project, Japan Science and Technology Agency, Saitama, Japan; 8 International Center for Medical Research and Treatment, Graduate School of Medicine, Kobe University, Chuo-ku, Hyogo, Japan; University of Maryland, United States of America

## Abstract

Currently, two neuraminidase (NA) inhibitors, oseltamivir and zanamivir, which must be administrated twice daily for 5 days for maximum therapeutic effect, are licensed for the treatment of influenza. However, oseltamivir-resistant mutants of seasonal H1N1 and highly pathogenic H5N1 avian influenza A viruses have emerged. Therefore, alternative antiviral agents are needed. Recently, a new neuraminidase inhibitor, R-125489, and its prodrug, CS-8958, have been developed. CS-8958 functions as a long-acting NA inhibitor *in vivo* (mice) and is efficacious against seasonal influenza strains following a single intranasal dose. Here, we tested the efficacy of this compound against H5N1 influenza viruses, which have spread across several continents and caused epidemics with high morbidity and mortality. We demonstrated that R-125489 interferes with the NA activity of H5N1 viruses, including oseltamivir-resistant and different clade strains. A single dose of CS-8958 (1,500 µg/kg) given to mice 2 h post-infection with H5N1 influenza viruses produced a higher survival rate than did continuous five-day administration of oseltamivir (50 mg/kg twice daily). Virus titers in lungs and brain were substantially lower in infected mice treated with a single dose of CS-8958 than in those treated with the five-day course of oseltamivir. CS-8958 was also highly efficacious against highly pathogenic H5N1 influenza virus and oseltamivir-resistant variants. A single dose of CS-8958 given seven days prior to virus infection also protected mice against H5N1 virus lethal infection. To evaluate the improved efficacy of CS-8958 over oseltamivir, the binding stability of R-125489 to various subtypes of influenza virus was assessed and compared with that of other NA inhibitors. We found that R-125489 bound to NA more tightly than did any other NA inhibitor tested. Our results indicate that CS-8958 is highly effective for the treatment and prophylaxis of infection with H5N1 influenza viruses, including oseltamivir-resistant mutants.

## Author Summary

Since the first human outbreak in Hong Kong in 1997, highly pathogenic H5N1 avian influenza A viruses have posed a threat to public health. Because some isolates exhibit resistance to oseltamivir, a WHO-recommended neuraminidase (NA) inhibitor for the treatment of H5N1 influenza infection, alternative antivirals are urgently needed. Here, we assessed the efficacy of CS-8958, a prodrug of the novel neuraminidase inhibitor R-125489, against highly pathogenic H5N1 influenza viruses in a murine lethal infection model. We found that CS-8958 confers more potent and long-lasting protection to mice against H5N1 influenza viruses, including oseltamivir-resistant mutants, than does oseltamivir. Further, we demonstrate that CS-8958 has substantial efficacy as both a therapeutic and a prophylactic agent against H5N1 influenza viruses in mice. CS-8958 is, therefore, a promising candidate antiviral for the prevention and treatment of influenza patients infected with H5N1 or other subtype viruses.

## Introduction

Human H1N1 and H3N2 influenza A viruses are highly contagious and cause “seasonal influenza” worldwide. The global impact of influenza epidemics is estimated to be 3.5 million cases of severe illness and 300,000 to 500,000 deaths annually [1]. The elderly, young children, and immunocompromised patients are particularly at risk, with substantial morbidity and mortality among these groups [2]. In addition, the emergence of a virus possessing hemagglutinin and neuraminidase (NA) to which humans have limited immunological memory creates the potential for “pandemic influenza”. In 1997, human infections with highly pathogenic H5N1 avian influenza viruses were first documented in Hong Kong [3]–[5]. Since then, these viruses have spread throughout Asia, Europe, and Africa with high morbidity and mortality among avian species and with occasional transmission to humans with high mortality (http://www.who.int/csr/disease/avian_influenza/en/). Although human-to-human transmission is rare, once the H5N1 viruses acquire this ability, a devastating pandemic may be inevitable.

Two countermeasures are available to control human influenza: vaccination and antiviral treatment. Although vaccination plays a critical role in influenza prophylaxis, it takes more than six months to produce sufficient vaccine to cover a large proportion of the human population upon the emergence of a new strain [6]. Therefore, antivirals are important tool to mitigate an influenza pandemic.

Currently, two types of anti-influenza drug are available: M2 ion channel blockers (amino-adamantines; amantadine and rimantadine) [7] and NA inhibitors (oseltamivir and zanamivir) [8]. However, amino-adamantine-resistant viruses readily emerge and are already prevalent worldwide among the seasonal influenza viruses (both H1N1 and H3N2 subtypes [9],[10]). In fact, the recently emerged swine-origin pandemic (H1N1) 2009 virus is already amino-adamantine-resistant [11]. Moreover, the emergence of amino-amantadine-resistant H5N1 viruses in Vietnam, Cambodia, and Thailand [12] has prompted the World Health Organization to recommend oseltamivir for the treatment and prophylaxis of human H5N1 influenza virus infections [13]. Accordingly, many countries have stockpiled oseltamivir in anticipation of an H5N1 pandemic.

NA inhibitor-resistant viruses were thought to not readily emerge, yet studies have demonstrated a higher prevalence of oseltamivir-resistant viruses than was expected among oseltamivir-treated patients [14],[15]. Person-to-person transmission of oseltamivir-resistant influenza B virus has been reported [16]. Moreover, oseltamivir-resistant H1N1 viruses were isolated in Europe during the 2007–2008 season [17] and are now widely circulating [18] (http://www.who.int/csr/disease/influenza/h1n1_table/en/index.html). Oseltamivir-resistant H5N1 viruses have been isolated from patients in Vietnam and Egypt [19],[20] (http://www.emro.who.int/csr/media/pdf/ai_press_22_01_07.pdf), some of whom died despite early initiation of drug treatment, suggesting that the resistant variants are just as virulent as their sensitive counterparts. These epidemics of oseltamivir-resistant influenza viruses, therefore, necessitate the development of alternative antiviral agents.

In response to the need for new anti-influenza drugs, CS-8958, a prodrug of the novel neuraminidase inhibitor R-125489, has been developed [21]. R-125489 inhibits the NA activity of various influenza A and B viruses *in vitro*, including N1-N9 subtypes and oseltamivir-resistant viruses, with limited cytotoxicity [21]. Further, a single dose of CS-8958 prolonged the survival of mice infected with a mouse-adapted A/Puerto Rico/8/34 (H1N1) [21]. Recently, we demonstrated the therapeutic efficacy of CS-8958 in mice infected with the swine-origin pandemic (H1N1) 2009 virus [22]. However, its efficacy against H5N1 influenza viruses, whose pathogenicity is substantially higher than that of seasonal mouse-adapted human influenza and swine-origin pandemic (H1N1) 2009 viruses [23], has not been assessed *in vivo*.

Here, we examined the efficacy of CS-8958 against H5N1 influenza viruses *in vitro* and *in vivo*. The binding stability of R-125489 to H1N1, H3N2, and type B influenza viruses was also assessed. We demonstrate the potential of CS-8958 as an alternative antiviral against influenza viruses, including oseltamivir-resistant mutants.

## Methods

### Viruses and cells

H5N1 influenza viruses A/Hanoi/30408/05 clone7 (HN30408cl7;; oseltamivir-sensitive) and clone9 and clone3 (oseltamivir-resistant) possessing a histidine-to-tyrosine substitution at position 274 (H274Y) and an asparagine-to-serine substitution at position 294 (N294S) in NA, respectively [20], and A/Indonesia/UT3006/05 (Ind3006) were isolated in Madin-Darby canine kidney (MDCK) cells. A/Vietnam/1203/04 (H5N1; VN1203) was generated in 293T cells by reverse genetics as described below. HN30408 and VN1203 are categorized as clade 1 viruses [24], whereas Ind3006 is in clade 2.1.3 [25]. Influenza viruses A/New Caledonia/20/99 (H1N1), A/Panama/2007/99 (H3N2), and B/Mie/1/93 were provided by the National Institute of Infectious Diseases, Japan. MDCK and 293T cells were maintained in Eagle's minimal essential medium (MEM) supplemented with 5% newborn calf serum (Sigma, St. Louis, MO) and in Dulbecco's modified Eagle's medium (Sigma) supplemented with 10% fetal calf serum, respectively. Both cell lines were cultured at 37°C in 5% CO_2_. All experiments with H5N1 viruses were performed in a biosafety level 3 containment laboratory inspected by the Ministry of Agriculture, Forestry and Fisheries, Japan.

### Compounds

CS-8958, R-125489 (the active metabolite of CS-8958), zanamivir and peramivir were synthesized according to published procedures [26]–[28]. Oseltamivir carboxylate (the active metabolite of oseltamivir phosphate) was prepared from oseltamivir phosphate extracted from Tamiflu® (Roche Laboratories Inc., Basel, Switzerland).

### Reverse genetics

Plasmids for the expression of VN1203 viral RNAs were constructed as described previously [29]. The H274Y and N294S mutations were independently introduced into the plasmid for the VN1203 NA gene. Wild-type and oseltamivir-resistant VN1203 (VN1203-H274Y and VN1203-N294S) were also generated by reverse genetics, as described previously [30], propagated in MDCK cells twice, and stored at −80°C as stock viruses. All constructed plasmids were sequenced to ensure the absence of unwanted mutations.

### NA activity inhibition assay

NA sensitivity to the NA inhibitors was evaluated by using an NA activity inhibition assay based on the method of Gubareva et al [31]. Methylumbelliferyl-N-acetylneuraminic acid (MUNANA, Sigma), at a final concentration of 0.1 mM, was used as the fluorescent substrate. Diluted virus containing 800 to 1200 fluorescence units of MUNANA was mixed with the test compound (0.01 nM-1 mM) in 33 mM 2-[N-morpholino]ethanesulfonic acid (pH 6.0) containing 4 mM CaCl_2_ and incubated for 30 min at 37°C. After adding the substrate and incubating at 37°C for 1 h, the reaction was stopped by adding 0.1M NaOH in 80% ethanol (pH 10.0). Fluorescence was measured at an excitation wavelength of 360 nm and an emission wavelength of 465 nm. The relationship between the concentration of inhibitor and the percentage of fluorescence inhibition was determined and 50% inhibitory concentration (IC_50_) values were obtained by extrapolating those findings.

### Therapeutic efficacy of NA inhibitors in mice

Six-week-old female BALB/c mice (Japan SLC Inc., Shizuoka, Japan) were anesthetized by sevoflurane inhalation and intranasally infected with 4 MLD_50_ (50% mouse lethal dose) of viruses in 50 µl of phosphate-buffered saline (PBS). Eight or ten mice per group were given CS-8958 (75, 750, or 1,500 µg/kg) 2 h post-infection (pi) intranasally or oseltamivir phosphate (5 or 50 mg/kg twice a day for 5 days or 10 or 100 mg/kg once 2 h pi) orally. Control mice for the CS-8958 and oseltamivir phosphate groups received saline intranasally and water orally, respectively. Mouse survival was monitored daily for 21 days after virus infection.

On days 3 and 6 pi, three mice per group were euthanized for virologic examinations. To determine the virus titers in lungs and brain, each organ was homogenized in PBS containing antibiotics. Cellular debris was removed by centrifugation at 3,000×g for 10 min, after which the supernatants were subjected to plaque assays in MDCK cells.

### Prophylactic efficacy of NA inhibitors in mice

Eight BALB/c mice per group were given CS-8958 (75, 750, or 1,500 &g/kg) intranasally or oseltamivir phosphate (10 or 100 mg/kg) orally 7, 3, or 1 day before or 2 h after infection with 4 MLD_50_ of HN30408cl7. Mouse survival was monitored daily for 21 days after virus infection. As above (except for the 10 mg/kg oseltamivir phosphate), we also determined the virus titers in the lungs and brain of three mice per group on days 3 and 6 pi.

### Binding stability of NA inhibitors

To assess the binding stability of NA inhibitors to viral NAs, we measured the NA activity of viruses treated with excess NA inhibitors according to a previous report [32]. Briefly, A/New Caledonia/20/99 (H1N1), A/Panama/2007/99 (H3N2), and B/Mie/1/93 were incubated with approximately 10–20 IC_90_ of R-125489, peramivir, zanamivir, or oseltamivir carboxylate at 37°C for 60–90 min. Unbound NA inhibitors were removed from the drug-virus mixtures with a Bio-Spin P-6 column (Bio-Rad Laboratories, Hercules, CA). Using 0.1 mM MUNANA (Nacalai Tesque, Inc., Kyoto, Japan) in 32.5 mM MES - 4 mM CaCl_2_ (pH 6.5) as a fluorescent substrate, we monitored the NA activity of the drug-bound viruses every 15 min for 6 h at room temperature by measuring the fluorescence at an excitation wavelength of 360 nm and an emission wavelength of 460 nm.

### Statistical analysis

Survival rates and median survival times of infected mice were calculated by the Kaplan-Meier method [33]. A log-rank test based on a joint ranking method was used to compare the compound groups with the saline group. The analyses were performed using the SAS System Release 8.2 for Windows (SAS Institute, Inc., Cary, NC). P values less than 0.05 were considered to be statistically significant.

### Ethics

Our research protocol with mice followed the Regulations for Animal Care and Use of the University of Tokyo and was approved by the Animal Experiment Committee of the Institute of Medical Science, the University of Tokyo (approval number: 19–29).

## Results

### Inhibitory effect of CS-8958 on the NA activity of H5N1 influenza viruses

To evaluate the efficacy of CS-8958 against H5N1 influenza viruses, NA activity in the presence of various NA inhibitors was measured *in vitro* (Table 1). The NA activity of the oseltamivir-sensitive H5N1 influenza virus A/Hanoi/30408/05 clone7 (HN30408cl7) was inhibited by R-125489 (the active form of CS-8958), oseltamivir carboxylate (the active form of oseltamivir), and zanamivir, whereas that of the oseltamivir-resistant variants HN30408 clone9 and clone3, which possess a histidine-to-tyrosine substitution at position 274 (H274Y) and an asparagine-to-serine substitution at position 294 (N294S) in NA, respectively [20], was reduced by R-125489 and zanamivir, but not by oseltamivir carboxylate. Similarly, wild-type A/Vietnam/1203/04 (H5N1; VN1203) and its oseltamivir-resistant mutants were sensitive to R-125489 and zanamivir at similar levels, whereas the sensitivity of VN1203-H274Y and VN1203-N294S to oseltamivir carboxylate was appreciably lower than that of the parent VN1203. Further, the NA activity of A/Indonesia/UT3006/05 (H5N1; Ind3006), a clade 2.1.3 virus, was also inhibited by oseltamivir carboxylate, zanamivir and R-125489. These results indicate that R-125489 and zanamivir effectively inhibit the NA activity of H5N1 viruses, including oseltamivir-resistant viruses and those of different clades.

**Table 1:** Inhibitory effect of R-125489, zanamivir, and oseltamivir carboxylate on the NA activity of H5N1 influenza viruses.

	IC_50_ (95% confidential interval)						
	HN30408			VN1203			Ind3006
	clone7	clone9 (H274Y^c^)	clone3 (N294S^c^)	Wild-type	H274Y	N294S	
R-125489^a^	0.32^d^ (0.18–0.56)	1.1 (0.84–1.3)	1.6 (0.84–2.4)	0.28 (0.27–0.29)	2.1 (1.4–3.0)	1.4 (0.48–2.6)	0.29 (0.24–0.35)
Zanamivir	0.72 (0.50–1.2)	0.68 (0.58–0.80)	0.57 (0.54–0.61)	0.15 (0.12–0.17)	0.22 (0.21–0.22)	0.48 (0.34–0.73)	0.070 (0.067–0.074)
Oseltamivir carboxylate^b^	0.35 (0.31–0.39)	430 (370–500)	1.6 (0.84–2.4)	0.31 (0.26–0.37)	1100 (570–1700)	28 (21–38)	10 (7.7–13)

a R-125489 is the active form of CS-8958.

b Oseltamivir carboxylate is the active form of oseltamivir.

c The H274Y and N294S mutations in NA confer oseltamivir-resistance to H5N1 influenza viruses [20].

d IC_50_ values: mean nmol/l of duplicate reactions.

### Therapeutic efficacy of CS-8958 against H5N1 influenza viruses in mice

**Figure 1:**
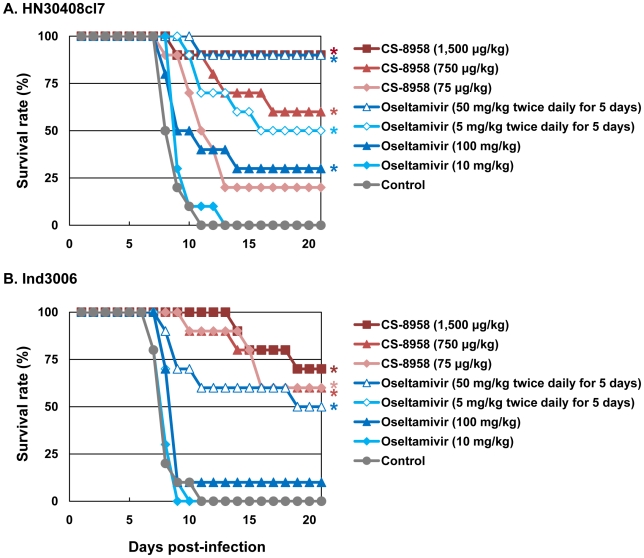
Therapeutic efficacy of CS-8958 and oseltamivir phosphate against H5N1 influenza viruses in mice. Ten mice per group were intranasally infected with 4 MLD_50_ of HN30408cl7 (A) or Ind3006 (B). The infected mice were given CS-8958 intranasally or oseltamivir phosphate orally at the indicated doses 2 h post-infection (pi) or twice daily for 5 days beginning 2 h pi. Control mice received saline and water 2 h pi. Survival was monitored daily for 21 days. *P* values were calculated by a log-rank test using a joint ranking method. Asterisk, *P*<0.05.

To evaluate the therapeutic efficacy of CS-8958 against H5N1 influenza viruses, we used a murine lethal infection model. Two hours post-infection (pi) with 4 MLD_50_ of HN30408cl7 or Ind3006, mice were given a single dose of CS-8958 intranasally or oseltamivir phosphate orally as either a single dose or twice daily for 5 days. Survival of infected mice was monitored daily for 21 days (Figure 1A and B). All the control mice died by 11 days pi. The single dose of oseltamivir phosphate protected less than half of the mice from lethal infection. However, the administration of 50 mg/kg of oseltamivir phosphate twice daily for 5 days saved 90% or 50% of mice infected with HN30408cl7 or Ind3006, respectively. Under these conditions, the single 1,500 µg/kg dose of CS-8958 protected 90% or 70% of mice from HN30408cl7 or Ind3006, respectively.

To further evaluate the therapeutic efficacy of CS-8958 against H5N1 viruses, we determined virus titers in lungs and brain of HN30408cl7- or Ind3006-infected mice on days 3, 6, and 9 pi (Table 2). CS-8958, at 750 or 1,500 µg/kg, dramatically inhibited the replication of both viruses, particularly in the brain. Interestingly, the efficacy of oseltamivir against Ind3006, which is oseltamivir-sensitive, was limited with respect to reducing virus titers in mouse organs, although its in vivo efficacy was evident (Figure 1B). These results indicate that CS-8958 confers more potent and long-lasting protection to mice against H5N1 influenza viruses than does oseltamivir phosphate.

**Table 2:** Virus titers in H5N1 virus-infected mice subsequently treated with drug.

Virus	Treatment		Virus titers (mean log_10_ PFU±SD/g) in:					
			Day 3		Day 6		Day 9	
			Lungs	Brain	Lungs	Brain	Lungs	Brain
HN30408cl7	Control		5.9±0.4	<1.6	5.8±1.1	4.6, 4.7	ND^a^	ND
	CS-8958	75 µg/kg	4.1, 5.0	<1.6	5.1±0.3	2.0±0.3	5.9±1.1	5.5±0.4
		750 µg/kg	4.1, 4.5	<1.6	4.0, 4.3	<1.6	2.7±1.5	<1.6
		1,500 µg/kg	2.0	<1.6	<1.7	<1.6	3.0, 3.5	<1.6
	Oseltamivir phosphate	10 mg/kg	6.2±0.2	<1.6	6.4±0.6	4.8±1.5	6.1	5.1
		100 mg/kg	6.0±0.1	<1.6	5.2±0.1	2.5, 2.5	4.9±1.8	4.8
		5 mg/kg twice daily for 5 days	5.0±0.6	<1.6	5.5±0.4	2.9±0.4	4.7±0.6	4.7±0.4
		50 mg/kg twice daily for 5 days	5.0, 5.3	<1.6	3.8±0.5	<1.6	4.7±1.4	3.7
Ind3006	Control		7.2±0.1	<1.6	7.3±0.2	3.7±0.3	ND	ND
	CS-8958	75 µg/kg	6.4±0.2	<1.6	7.2±0.1	<1.6	<1.7	<1.6
		750 µg/kg	6.0, 6.0	<1.6	6.0, 6.9	<1.6	<1.7	<1.6
		1,500 µg/kg	2.4, 4.8	<1.6	4.5±1.4	<1.6	<1.7	<1.6
	Oseltamivir phosphate	10 mg/kg	7.5±0.1	<1.6	7.5±0.2	3.1±0.3	<1.7	<1.6
		100 mg/kg	7.2±0.1	<1.6	7.4±0.2	2.6±0.1	<1.7	<1.6
		5 mg/kg twice daily for 5 days	6.5, 7.2	<1.6	7.4±0.1	3.2±1.2	<1.7	<1.6
		50 mg/kg twice daily for 5 days	7.1±0.2	<1.6	7.2±0.1	3.3±0.9	<1.7	<1.6

Three mice per group were intranasally infected with 4 MLD_50_ of HN30408cl7 or Ind3006 and given CS-8958 intranasally or oseltamivir phosphate orally at the indicated doses 2 h post-infection (pi) or twice daily for 5 days beginning 2 h pi, respectively. Control mice received saline and water 2 h pi. When only one or two mice in a group were negative for virus, individual titers were recorded.

a ND: no data since all mice died by day 9.

### Therapeutic efficacy of CS-8958 against oseltamivir-resistant H5N1 influenza viruses in mice

**Figure 2:**
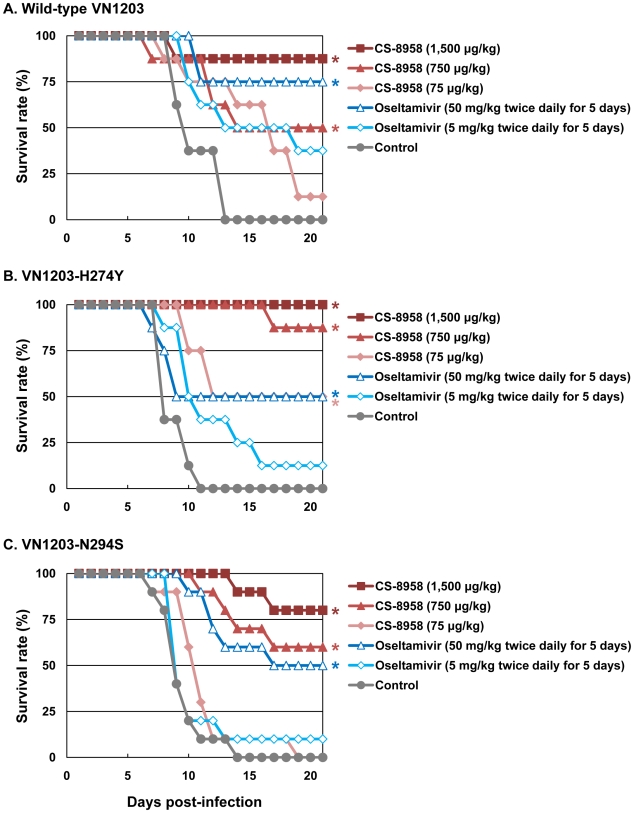
Therapeutic efficacy of CS-8958 and oseltamivir phosphate against oseltamivir-resistant H5N1 influenza viruses in mice. Eight mice per group were intranasally infected with 4 MLD_50_ of wild-type VN1203 (A), VN1203-H274Y (B), or VN1203-N294S (C). The infected mice were given CS-8958 intranasally or oseltamivir phosphate orally at the indicated doses 2 h post-infection (pi) or twice daily for 5 days beginning 2 h pi. Control mice received saline and water 2 h pi. Survival was monitored daily for 21 days. *P* values were calculated by a log-rank test using a joint ranking method. Asterisk, *P*<0.05.

To evaluate the therapeutic efficacy of CS-8958 against oseltamivir-resistant H5N1 influenza viruses, we used the oseltamivir-resistant H5N1 viruses VN1203-H274Y and VN1203-N294S. These mutant viruses (VN1203-H274Y and VN1203-N294S) are as virulent as their parental virus, as shown by their MLD_50_s: wild-type VN1203, 4.7 plaque-forming units (PFU); VN1203-H274Y, 2.1 PFU; and VN1203-N294S, 4.7 PFU. A single dose of CS-8958 was efficacious for mice infected with 4 MLD_50_ of VN1203-H274Y or VN1203-N294S, as well as wild-type VN1203 (Figure 2). Oseltamivir phosphate, however, was less effective against these two mutant viruses, as expected. Our data thus demonstrate the therapeutic efficacy of CS-8958 against oseltamivir-resistant H5N1 influenza viruses in mice.

### Prophylactic efficacy of CS-8958 against H5N1 influenza viruses in mice

**Figure 3:**
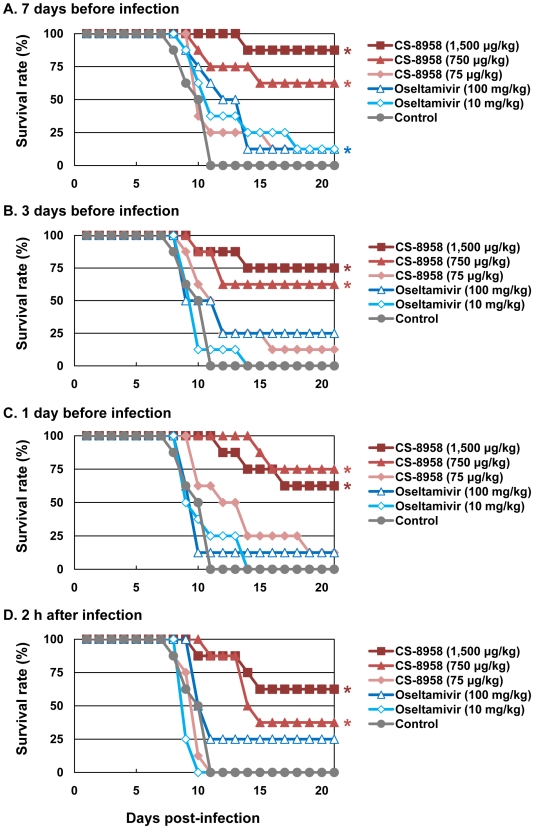
Prophylactic effect of CS-8958 and oseltamivir phosphate against H5N1 influenza viruses in mice. Eight mice per group were given CS-8958 intranasally or oseltamivir phosphate orally at the indicated doses on day 7 (A), 3 (B) or 1 (C) before or 2 h (D) after infection with 4 MLD_50_ of HN30408cl7. Control mice received saline and water 2 h post-infection. Survival was monitored daily for 21 days. *P* values were calculated by a log-rank test using a joint ranking method. Asterisk, *P*<0.05.

To evaluate the prophylactic efficacy of CS-8958 against H5N1 influenza viruses, we administrated HN30408cl7-infected mice with CS-8958 intranasally or oseltamivir phosphate orally 7, 3, or 1 day before or 2 h after virus infection. The single CS-8958 dose of 750 or 1,500 µg/kg significantly protected mice from the lethal infection even in mice who received the drug 7 days prior to infection (*p* = 0.0029 and <0.0001, respectively). The prophylactic efficacy of 75 µg/kg of CS-8958 and that of 10 or 100 mg/kg of oseltamivir phosphate was, however, limited (Figures 3A to D).

To further evaluate the prophylactic efficacy of CS-8958 against H5N1 viruses, we determined the virus titers in the lungs and brain of the infected mice on days 3, and 6 pi (Table 3). Compared to oseltamivir-treated mice, those treated with 750 or 1,500 µg/kg of CS-8958 exhibited lower levels of virus in the brain. In the lungs on day 3 pi, virus titers were at least one log lower in animals treated with 750 or 1,500 µg/kg of CS-8958 compared to those treated with oseltamivir, although virus titers in the lungs on day 6 were only somewhat lower (less than one log in some cases) in mice treated with 750 or 1,500 µg/kg of CS-8958 compared to oseltamivir-treated mice. These results suggest that CS-8958 may be useful as both a therapeutic and a prophylactic drug against H5N1 influenza viruses.

**Table 3:** Prophylactic efficacy of CS-8958 as measured by H5N1 virus growth in mice.

Drug given at:	Treatment		Virus titer (mean log_10_ PFU±SD/g) in:			
			Day 3		Day 6	
			Lungs	Brain	Lungs	Brain
+2 h	Control		6.0±0.2	<1.6	6.6±1.0	4.6±1.1
−7 days	CS-8958	75µg/kg	5.6±0.3	<1.6	6.7±0.3	1.9±1.7
		750µg/kg	4.5±0.6	<1.6	6.3±0.2	4.0
		1500µg/kg	4.5±0.3	<1.6	5.0±0.4	<1.6
	Oseltamivir phosphate	100mg/kg	5.8±0.8	<1.6	6.0±0.4	1.6, 2.4
−3 days	CS-8958	75µg/kg	5.4±0.2	<1.6	6.2±0.2	<1.6
		750µg/kg	4.7±0.3	<1.6	4.8±0.1	<1.6
		1500µg/kg	4.1±0.6	<1.6	4.1±1.3	<1.6
	Oseltamivir phosphate	100mg/kg	5.5±0.7	<1.6	5.6±0.4	2.4, 2.8
−1 day	CS-8958	75µg/kg	3.2±2.8	<1.6	5.6±0.4	<1.6
		750µg/kg	3.5±0.8	<1.6	4.7±1.0	<1.6
		1500µg/kg	2.9±0.3	<1.6	4.7±1.2	<1.6
	Oseltamivir phosphate	100mg/kg	4.3±1.9	<1.6	6.1±0.2	2.6
+2 h	CS-8958	75µg/kg	5.1±0.2	<1.6	5.8±0.6	<1.6
		750µg/kg	3.9±0.6	<1.6	5.5±1.5	<1.6
		1500µg/kg	1.4±2.5	<1.6	3.6±0.8	<1.6
	Oseltamivir phosphate	100mg/kg	3.2±2.9	<1.6	6.0±0.0	<1.6

Three mice per group were intranasally infected with 4 MLD_50_ of HN30408cl7 and given CS-8958 intranasally or oseltamivir phosphate orally at the indicated doses at the indicated time points. Control mice received saline and water. When only one or two mice in a group were negative for virus, individual titers were recorded.

### Binding stability of R-125489 to viruses

**Figure 4:**
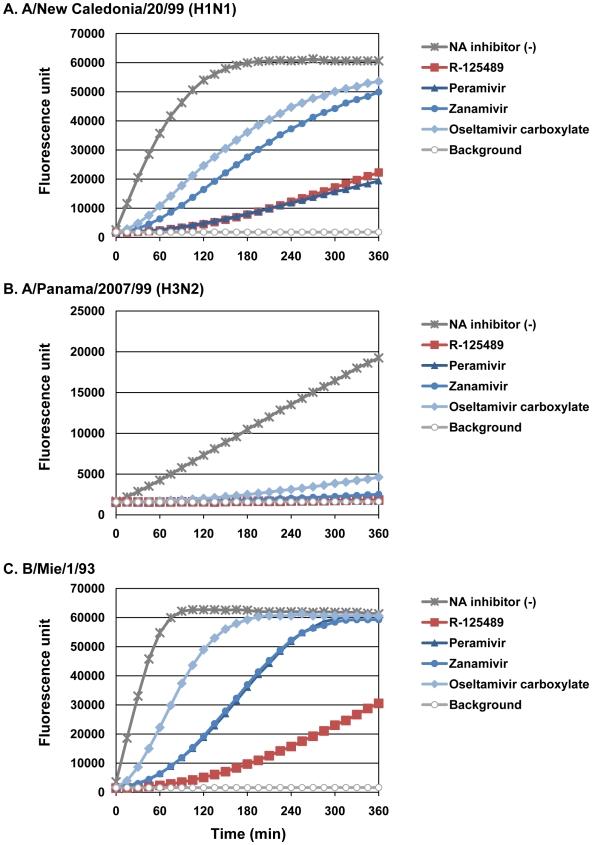
Binding stability of NA inhibitors to viruses. The NA activity of A/New Caledonia/20/99 (H1N1; A), A/Panama/2007/99 (H3N2; B), and B/Mie/1/93 (C) untreated [NA inhibitor (-)] or treated with excess R-125489, peramivir, zanamivir, or oseltamivir carboxylate, was monitored every 15 min for 6 h.

Bantia et al. [32] demonstrated that peramivir, a selective NA inhibitor [28],[34],[35], binds to NA more tightly than oseltamivir carboxylate or zanamivir by treating purified N9 NA with excess (100 nM) NA inhibitors, removing the unbound NA inhibitors and then measuring the NA activity over time. To assess the binding stability of R-125489 to virus NA, we performed similar experiments with the whole influenza virions A/New Caledonia/20/99 (H1N1), A/Panama/2007/99 (H3N2), and B/Mie/1/93 instead of purified NA; the NA activity of these viruses treated with excess R-125489, peramivir, zanamivir, or oseltamivir carboxylate was measured (Figure 4 and Tables S1 and S2).

We found a considerable difference in the dissociation rates of the NA inhibitors among our test virus strains. For example, the NA activity of A/Panama/2007/99 (H3N2) was severely inhibited by all test NA inhibitors for at least 5 h, whereas the NA activity of B/Mie/1/93 treated with oseltamivir carboxylate was almost fully recovered by 3 h after substrate addition. Under these conditions, R-125489 appreciably inhibited the NA activity of A/New Caledonia/20/99 (H1N1) and A/Panama/2007/99 (H3N2) at a level similar to that of peramivir. Further, it inhibited the NA activity of B/Mie/1/93 by about 50%, even 6 h after substrate addition, whereas no inhibitory effect was observed at this time point for the other three NA inhibitors. These results suggest that R-125489 bound to viral NA more stably than did any other NA inhibitor tested.

## Discussion

This is the first study to demonstrate that CS-8958 is effective against H5N1 influenza viruses, including oseltamivir-resistant mutants, *in vitro* and *in vivo*. In addition, our study reveals the tight binding of R-125489 to NA. Previous animal studies suggest that increased or extended doses of oseltamivir phosphate are needed to protect mice and ferrets against recent H5N1 strains, which are more virulent than their predecessors [36]. Moreover, recent Indonesian clade 2 H5N1 isolates have reduced sensitivity to oseltamivir [37]. Here, we showed that CS-8958 inhibits the NA activity of oseltamivir-sensitive and -resistant H5N1 viruses *in vitro* (Table 1). In mice, CS-8958 exhibited a strong and lasting therapeutic effect against both oseltamivir-sensitive and -resistant H5N1 viruses (Figures 1 and 2). These results clearly indicate that CS-8958 is effective even against H5N1 virus strains to which the efficacy of oseltamivir phosphate is limited.

To further evaluate the therapeutic efficacy of CS-8958 against H5N1 viruses, we determined virus titers in lungs and brain of HN30408cl7- or Ind3006-infected mice on days 3, 6, and 9 pi (Table 2). CS-8958, at 750 or 1,500 µg/kg, dramatically inhibited the replication of both viruses, particularly in the brain. These results indicate that CS-8958 confers more potent and long-lasting protection to mice against H5N1 influenza viruses than does oseltamivir phosphate.

We also observed a prophylactic effect of CS-8958 against H5N1 viruses in mice (Figure 3). Similar efficacy was demonstrated in mice infected with a mouse-adapted A/Puerto Rico/8/34 [21]. Although studies in higher mammals (e.g., ferrets and macaques) are required, this noteworthy feature of CS-8958 may prove to be an important differentiator with respect to other NA inhibitors (i.e., oseltamivir phosphate, zanamivir, and peramivir).

The long-lasting therapeutic and prophylactic efficacy of intranasally-administrated CS-8958 in mice was previously thought to be due to its rapid metabolism to R-125489 and the retention of the metabolite in the lungs [21] (Koyama et al., unpublished data). However, peramivir, which exhibits significant therapeutic efficacy in mice following a single intramuscular injection, was shown to bind to N9 NA more tightly than oseltamivir carboxylate or zanamivir [32]. Here, we revealed that the binding stabilities of R-125489 to H1N1 and H3N2 viruses are comparable to that of peramivir and are superior to that of peramivir for type B virus (Figure 4). These findings suggest that the tight binding to NA may also account for the long-lasting therapeutic and prophylactic effects of CS-8958 *in vivo*.

Importantly, a single dose of CS-8958 conferred a more potent and long-lasting protective effect to mice against H5N1 influenza viruses than that of oseltamivir phosphate. CS-8958 is, therefore, a promising candidate for a new neuraminidase inhibitor to prevent and treat influenza patients infected with H5N1 and other subtype viruses.

## Supporting Information

Table S1 Slopes of the dissociation curve^a^. ^a^Slopes of the dissociation curve for each compound were estimated based upon the fluorescence from 0 to 50000 by a linear regression analysis assuming the intercepts equal to 0.
(0.04 MB DOC)

Table S2 Statistical analysis of slopes^a^. ^a^The slope difference and the 95% confidential interval (CI) between the slopes of two compound were compared and the corresponding P values were calculated. ^b^The numbers represent each compound as follows; 1: R-125489, 2: Zanamivir, 3: Oseltamivir carboxylate, 4: Peramivir, 5: Background.
(0.05 MB DOC)
