# Supplementary material for: Efficacy of the New Neuraminidase Inhibitor CS-8958 against H5N1 Influenza Viruses
Source: PLoS Pathog. 2010 Feb 26;6(2):e1000786. doi: 10.1371/journal.ppat.1000786 (PMC2829070; doi:10.1371/journal.ppat.1000786)
Supplement: Table S1 — Slopes of the dissociation curvea. aSlopes of the dissociation curve for each compound were estimated based upon the fluorescence from 0 to 50000 by a linear regression analysis assuming the intercepts equal to 0. (0.04 MB DOC) [file ppat.1000786.s001.doc]

**Supporting Table 1. Slopes of the dissociation curve^a^.**

| Virus | Compound | Slope of the dissociation curve |
| --- | --- | --- |
| A/New Caledonia/20/99  (H1N1) | R-125489 | 47.44 |
|  | Zanamivir | 140.10 |
|  | Oseltamivir carboxylate | 173.34 |
|  | Peramivir | 43.10 |
|  | Background | 499.62 |
| A/Panama/2007/99 (H3N2) | R-125489 | 0.65 |
|  | Zanamivir | 2.10 |
|  | Oseltamivir carboxylate | 6.97 |
|  | Peramivir | 0.19 |
|  | Background | 48.99 |
| B/Mie/1/93 | R-125489 | 65.56 |
|  | Zanamivir | 184.10 |
|  | Oseltamivir carboxylate | 381.21 |
|  | Peramivir | 180.66 |
|  | Background | 954.08 |

^a^Slopes of the dissociation curve for each compound were estimated based upon the fluorescence from 0 to 50000 by a linear regression analysis assuming the intercepts equal to 0.
