# Supplementary material for: Efficacy of the New Neuraminidase Inhibitor CS-8958 against H5N1 Influenza Viruses
Source: PLoS Pathog. 2010 Feb 26;6(2):e1000786. doi: 10.1371/journal.ppat.1000786 (PMC2829070; doi:10.1371/journal.ppat.1000786)
Supplement: Table S2 — Statistical analysis of slopesa. aThe slope difference and the 95% confidential interval (CI) between the slopes of two compound were compared and the corresponding P values were calculated. bThe numbers represent each compound as follows; 1: R-125489, 2: Zanamivir, 3: Oseltamivir carboxylate, 4: Peramivir, 5: Background. (0.05 MB DOC) [file ppat.1000786.s002.doc]

**Supporting Table 2. Statistical analysis of slopes^a^.**

| Virus | Comparison^b^ | Slope difference^b^ | P value | 95% CI | |
| --- | --- | --- | --- | --- | --- |
|  |  |  |  | Lower | Upper |
| A/New Caledonia/20/99  (H1N1) | 1-5 | -452.18 | <0.0001 | -478.95 | -425.42 |
|  | 2-5 | -359.52 | <0.0001 | -385.70 | -333.34 |
|  | 3-5 | -326.28 | <0.0001 | -355.89 | -296.66 |
|  | 4-5 | -456.52 | <0.0001 | -478.23 | -434.81 |
|  | 1-4 | 4.34 | 0.082 | -0.57 | 9.25 |
|  | 2-4 | 97.00 | <0.0001 | 92.21 | 101.80 |
|  | 3-4 | 130.25 | <0.0001 | 124.36 | 136.13 |
|  | 1-3 | -125.91 | <0.0001 | -132.69 | -119.12 |
|  | 2-3 | -33.24 | <0.0001 | -39.92 | -26.57 |
|  | 1-2 | -92.66 | <0.0001 | -98.28 | -87.05 |
| A/Panama/2007/99 (H3N2) | 1-5 | -48.35 | <0.0001 | -48.73 | -47.96 |
|  | 2-5 | -46.89 | <0.0001 | -47.32 | -46.46 |
|  | 3-5 | -42.02 | <0.0001 | -42.71 | -41.34 |
|  | 4-5 | -48.81 | <0.0001 | -49.19 | -48.42 |
|  | 1-4 | 0.46 | <0.0001 | 0.40 | 0.52 |
|  | 2-4 | 1.92 | <0.0001 | 1.71 | 2.12 |
|  | 3-4 | 6.78 | <0.0001 | 6.21 | 7.36 |
|  | 1-3 | -6.32 | <0.0001 | -6.90 | -5.75 |
|  | 2-3 | -4.87 | <0.0001 | -5.47 | -4.26 |
|  | 1-2 | -1.46 | <0.0001 | -1.67 | -1.25 |
| B/Mie/1/93 | 1-5 | -888.52 | <0.0001 | -998.79 | -778.24 |
|  | 2-5 | -769.97 | <0.0001 | -927.51 | -612.43 |
|  | 3-5 | -572.87 | <0.0001 | -667.99 | -477.74 |
|  | 4-5 | -773.42 | <0.0001 | -926.42 | -620.41 |
|  | 1-4 | -115.10 | <0.0001 | -131.03 | -99.17 |
|  | 2-4 | 3.44 | 0.78 | -21.20 | 28.08 |
|  | 3-4 | 200.55 | <0.0001 | 159.64 | 241.46 |
|  | 1-3 | -315.65 | <0.0001 | -345.46 | -285.84 |
|  | 2-3 | -197.10 | <0.0001 | -239.03 | -155.18 |
|  | 1-2 | -118.54 | <0.0001 | -134.74 | -102.35 |

^a^The slope difference and the 95% confidential interval (CI) between the slopes of two compound were compared and the corresponding P values were calculated.

^b^The numbers represent each compound as follows; 1: R-125489, 2: Zanamivir, 3: Oseltamivir carboxylate, 4: Peramivir, 5: Background.
